# In-Vivo NMR Spectroscopy: A Powerful and Complimentary Tool for Understanding Environmental Toxicity

**DOI:** 10.3390/metabo8020035

**Published:** 2018-05-24

**Authors:** Monica Bastawrous, Amy Jenne, Maryam Tabatabaei Anaraki, André J. Simpson

**Affiliations:** 1 Department of Physical and Environmental Sciences, University of Toronto Scarborough, Toronto, ON M1C 1A4, Canada; monica.bastawrous@mail.utoronto.ca (M.B.); m.tabatabaeianaraki@mail.utoronto.ca (M.T.A.); 2 Department of Chemistry, University of Toronto Scarborough, Toronto, ON M1C 1A4, Canada; amy.jenne@mail.utoronto.ca

**Keywords:** metabolomics, environmental toxicology, in-vivo, NMR

## Abstract

Part review, part perspective, this article examines the applications and potential of in-vivo Nuclear Magnetic Resonance (NMR) for understanding environmental toxicity. In-vivo NMR can be applied in high field NMR spectrometers using either magic angle spinning based approaches, or flow systems. Solution-state NMR in combination with a flow system provides a low stress approach to monitor dissolved metabolites, while magic angle spinning NMR allows the detection of all components (solutions, gels and solids), albeit with additional stress caused by the rapid sample spinning. With in-vivo NMR it is possible to use the same organisms for control and exposure studies (controls are the same organisms prior to exposure inside the NMR). As such individual variability can be reduced while continual data collection over time provides the temporal resolution required to discern complex interconnected response pathways. When multidimensional NMR is combined with isotopic labelling, a wide range of metabolites can be identified in-vivo providing a unique window into the living metabolome that is highly complementary to more traditional metabolomics studies employing extracts, tissues, or biofluids.

## 1. Introduction

Anthropogenic activity since the beginning of the industrial revolution has had increasingly negative consequences on the environment, many of which are just beginning to be discovered, with some of the largest impacts on water, soil, and air quality [1]. Environmental contamination is a source of growing concern, with wide impacts and implications for both environmental and human health [2]. This manuscript is part review and part perspective with a focus on environmental toxicity that will provide an insight into current in-vivo Nuclear Magnetic Resonance (NMR) metabolomics approaches, as well as a discussion of the future potential.

### 1.1. Common Sources of Contamination

#### 1.1.1. Water Contamination

Clean water is a rapidly decreasing resource that threatens 1.1 billion people around the globe lacking sufficient access [3]. There are many sources of water contamination that are introduced through run-off from agriculture, disposal of personal care products, and industrial processes including heavy metal contamination [1]. Over the last 15 years, pharmaceuticals have been receiving increased attention as emerging contaminants in water bodies, as they have potential negative impacts on water quality and aquatic ecosystems due to the lack of regulation [4,5]. Pharmaceuticals persist in the environment in part due to their incomplete elimination through sewage treatment [5]. Current methods leave 60–90% of pharmaceuticals remaining in the water after treatment [4]. The concentrations and identity of many of these pharmaceuticals are just being discovered and even less is known about the impacts of these xenobiotics (and their transformation products) on the aquatic ecosystem, and human health [5]. Water pollution has become a major threat to ecosystem health; thus, further research into identifying which contaminants pose the largest threat, and their toxic mode of action, is needed to determine effects on individual organisms and populations [6,7].

#### 1.1.2. Soil Contamination

In addition to the aquatic pollution, soil is also a potential sink for contaminants. Often this is related to heavy metals from industry and mining, but can also include a range of organic xenobiotics such as agrochemicals, pharmaceuticals, and surfactants. These contaminants bind to the soil and often bioaccumulate in plants [8]. Therefore, it is important to study plants to obtain information on environmental contaminant impacts, which can potentially serve as an indicator, and even predictor prior to larger scale ecosystem shifts [9,10,11,12].

#### 1.1.3. Air and Atmospheric Pollution

Approximately 95% of the Earth’s atmosphere is in an 8–12 km range surrounding the earth, known as the troposphere [13]. The troposphere represents a delicate chemical balance that can be easily disrupted. One prominent example being the release of chlorofluorocarbons (CFCs) that can persist in the environment leading to the continual depletion of ozone [14,15]. Ozone is critically important as it represents a key source of hydroxyl radicals, which react rapidly with most air pollutants [16]. In addition, increased levels of acid rain can change the transport potential and transformation of contaminants with widespread implications for ecosystems and human respiratory health [17,18,19].

### 1.2. A Bottom-Up Approach

A traditional top-down approach which involves identifying all contaminants, their degradation products, and then assessing toxicities on an individual compound basis is extremely challenging given the complexity and dynamics of our environment. Conversely, a bottom-up approach focuses on the organisms themselves, and asks questions such as; is a population stressed?; what are the stressor/stressors causing the stress?; and which biochemical pathways are impacted by the stressor/stressors?

As toxic impacts manifest more rapidly in the metabolic profile, compared to the genome or proteome, the metabolome represents a key biological indictor of stress [20]. NMR spectroscopy due to its high resolution, ability to identify molecules de-novo, and non-destructive nature represents an ideal detector for metabolic profiles in-vivo.

#### Metabolomics

Metabolomics is the study of the biochemical changes occurring within an organism often in response to exposure to external stressors [21,22]. Although the first examples of metabolite profiling appeared in the literature in the 1950s [23], metabolomics is a relatively new field that has been increasing in popularity in recent years and has found applications across a number of disciplines including human and animal health [24], drug discovery [25], ecology [26], food chemistry [27], microbiology [23], and environmental monitoring [28,29]. Historically, metabolomics has been used as a method of examining the effects of drugs in the medical field [23]. However, since 2001 there has been an interest in environmental studies as a method to detect and explain toxicity [23,30]. Environmental metabolomics studies changes in the metabolite profile with changing environmental conditions [30], and have been used on a host of different species including: worms [31,32,33,34,35,36,37,38], *Drosophila melanogaster* [39], *Daphnia magna* [20,40,41,42,43,44,45], *Hyalella azteca* [46,47,48], *Caenorhabditis elegans* [49,50,51], rodents [52,53,54,55], and plants [56,57,58,59,60]. Species are often chosen either due to their susceptibility to external stressors or their abundance geographically. For example, earthworms are useful organisms as they will absorb chemicals both through their skin and via soil ingestion, making it possible to examine if different physical uptake routes alter the impact of contaminants [37,61,62,63,64]. Many metabolomics studies are conducted in-vitro which permits sensitive measurements but often involves sacrificing organisms or using extracted cells [65]. As such these methods provide a snapshot of the effects on the species, but lack the temporal resolution to resolve complex interconnected biochemical response pathways. Such information is critical as the biochemical pathways impacted, and their interconnectivities, describe how chemicals are toxic (toxic mode of action) and provide an insight into how the organism responds to the stressor (bioconversion, excretion, disease, adaptation, etc.) [66]. Due to the need to better understand toxic modes of action, there has been renewed interest in metabolomics studies to examine the effects of environmental contaminants on species in-vivo. For the purposes of this article in-vivo studies are defined as experiments that involve studies on the whole living organism rather than a sub component of, or extraction from, an organism. Methods have recently been developed to keep organisms alive during metabolomics experiments, allowing for longer testing, as well as long-term effects to be studied [67]. Many of these in-vivo studies examine *Daphnia magna* as a model organism for aquatic toxicity testing. *D. magna* have been used as model organisms since the 1960s due to their ability to survive in a wide range of habitats, ease of maintenance to culture, and having a relatively short life cycle (~40 days) [68]. These species are considered a keystone species in the environment as they are a primary consumer of phytoplankton and a key food source for secondary consumers thus representing a critical link in many ecosystems [69,70,71]. They cannot produce essential lipids themselves and instead assimilate them from their diet (i.e., algae), which in turn are utilized by higher order predators. In-vivo metabolomics approaches represent a powerful tool to study these transfers from algae and assimilation into *D. magna* and how external stressors impact their biochemical function [72,73]. The potential of in-vivo NMR to study this, and processes in other species will be discussed in more depth in the “Why in-vivo NMR?” section.

### 1.3. Toxicity Today

Present environmental policies are set primarily based on acute toxicity of individual species monitored mainly through death and reproduction. The Environmental Protection Agency has created standardized protocols to determine the toxicity of various contaminants [74]. These protocols are created by determining the Lethal Concentration 50% (LC_50_), which is the concentration of the contaminant at which 50% of the organisms die, or the Lethal Dose 50% (LD_50_) which is a single dose that kills 50% of all the organisms [75]. These methods are an excellent first defense to identify acutely toxic chemicals quickly, but results are often variable between species and there is no information regarding the metabolic impacts, sub-lethal impacts, or bioaccumulation [65,76].

While these methods have provided key “front-line” information on acute toxicity, the ever-increasing number of contaminants and complex mixtures at sub-lethal levels require the development of complimentary methods. A 2007 report by the National Academy of Sciences proposed numerous changes, with a large emphasis on examining the effects from a biochemical perspective. They argued this entails examining the mode of action, bioaccumulation, and biochemical impacts to the metabolome of the organisms, not only over 48 h, but also over longer periods, and after exposure to examine recovery times [65,76]. NMR spectroscopy due to its non-invasive nature is ideally suited to provide in-depth metabolic information in-vivo to better understand the sub-lethal toxicity of individual chemicals and mixtures, providing information on the mode of action, bioaccumulation, biotransformation, molecular reactivity, excretion, binding and bioavailability of chemicals [67].

### 1.4. NMR Spectroscopy as an Environmental Tool

Nuclear Magnetic Resonance (NMR) Spectroscopy is one of the most powerful analytical tools in modern research, providing unprecedented levels of molecular information on chemical structure and inter/intra molecular interactions [67], with minimum sample preparation. NMR targets magnetically susceptible nuclei such as ^1^H, ^31^P, ^15^N, and ^13^C, and after excitation using radio frequencies inside a magnetic field, every unique chemical environment within a molecule gives rise to a signal [77]. ^1^H NMR has a high natural abundance and one of the highest gyromagnetic ratios (ratio of its magnetic moment to its angular momentum) resulting in high sensitivity. As such, 1-dimensional (1D) proton NMR is the most commonly used nuclei for metabolomics studies [78]. However, 2D NMR examining the correlation between ^1^H-^1^H and ^1^H-^13^C offers additional spectral dispersion for molecular fingerprinting and connectivity information for molecular assignment [79]. NMR is a tool that can be used with varying amounts of sample (even down to sub-nL eggs) [80,81,82], is a non-destructive technique, and is highly reproducible across samples and labs [83,84,85,86]. NMR is also fully quantitative and when applied appropriately, each nuclei gives the same response in the spectrum leading to accurate quantitation of unknowns without the need for internal standards [87,88,89,90,91,92,93,94]. This makes the technique attractive for many metabolomics studies. Similarly, NMR is excellent for kinetic studies to measure chemical reaction rates and biological processes [95,96].

NMR is highly versatile and can be used to study solid, liquid, and gel samples. Recently, a new technique termed comprehensive multiphase (CMP) NMR was introduced which can examine all three states simultaneously. As such providing the ability to examine the interactions between phases, organization, layering, and transport across phases in close to real-time [46,66,97,98].

After the development of wide-bore superconducting magnets, NMR has become widely applied in the clinical field in the form of Magnetic Resonance Imaging (MRI) [99]. In addition to imaging, MRI can also provide localized spectroscopy within larger species, although magnetic susceptibility distortions limit the amount of metabolic information that can be extracted [100]. Readers interested on the applications of MRI in the medical field may consult these reviews [99,101]. This article will not cover the applications of in-vivo MRI but instead will focus on the use of high field NMR spectrometers to provide high resolution in-vivo metabolic information on small organisms in relation to environmental toxicity.

Due to its many advantages, NMR has huge potential in environmental research [102]. Unlike most analytical approaches, it can be applied in-vivo and represents an effective and reproducible method of determining which contaminants in the environment have deleterious effects on organisms. NMR can also serve as a powerful tool in providing in-depth information on the metabolic responses of plants [60,103,104,105,106] and animals [38,53,107,108], while providing information on the toxic mode of action of contaminants in the environment. Considering the broader picture, the subtle effects at sub-lethal levels are arguably more hazardous to animal, plant, and human populations, as they are often detected too late, and only after physical symptoms become widespread. As such the development of NMR approaches to detect and explain sub-lethal stress are of paramount importance to protecting both environmental and human health from a continuum of evolving environmental stressors.

## 2. Types of NMR

### 2.1. Solution-State NMR

Several types of NMR technology are available for the in-vivo study of environmental samples. Among which, solution-state provides the highest resolution and most comprehensive molecular information for soluble components [66]. Multiple nuclei can be studied using in-vivo solution-state NMR, with ^1^H and ^31^P being the two most commonly studied nuclei to date in an environmental context. Phosphorous is convenient as it is a spin half nucleus (i.e., produces sharp lines) and is relatively abundant (can be studied without enrichment) [109]. This nucleus is present in key bioenergetics molecules such as ATP/ADP and in DNA/RNA bases, both categories being important indicators of stress, such as changes in the energy cycle or DNA/RNA oxidation [110,111]. Proton NMR is also commonly studied due to its natural high abundance, its occurrence in most metabolites, and the availability of ^1^H NMR metabolic databases for assignments [24,78,112].

#### 2.1.1. In-Vivo Applications

Early studies employed ^31^P NMR spectroscopy for pioneering in-vivo analysis for the characterization of embryogenesis in plaice and freshwater catfish [113,114]. NMR experiments looking at phosphorous in larger organisms, such as Atlantic Cod (~40 cm) provided important information on temperature-dependent changes in the metabolome [115]. In this later case, while 1D NMR spectra provided metabolic discrimination, an MRI system was used to accommodate the larger organisms. Early pioneering of in-vivo analysis, also employed ^1^H NMR, where valuable information on hypoxic stress in marine worms was obtained [116].

Key applications of solution-state in-vivo NMR experiments involve the integration of flow systems for aquatic organisms. The use of a flow system in NMR experiments can be seen as early as 1981 to study metabolite responses to cadmium in *Chironomus tentans* and *D. magna*, which demonstrated the potential of flow in-vivo NMR to study metabolism [110].

**Figure 1:**
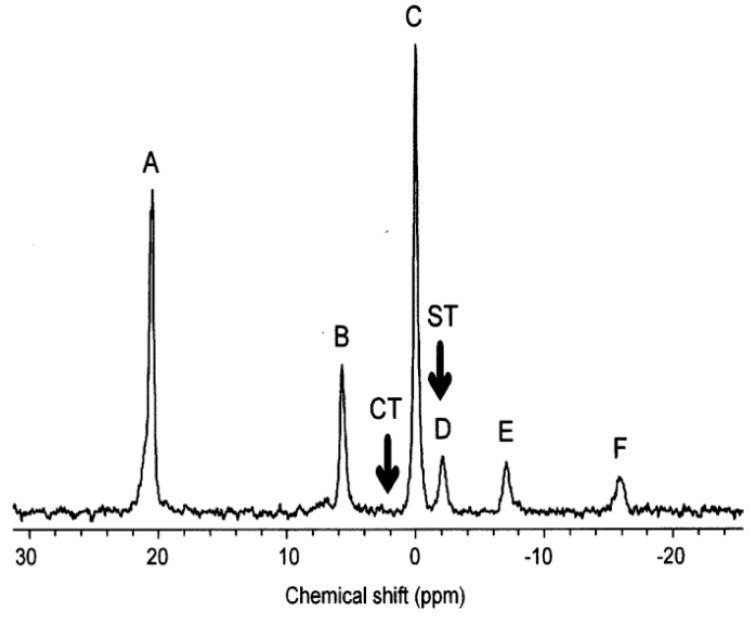
An example of an early NMR exposure study. The ^31^P NMR spectrum of molluscs (mussels) during acute exposure to copper. The assigned peaks are: A, MDP external standard; B, inorganic phosphate; C, phosphoarginine; D, overlapping γ-ATP and β-ADP; E, overlapping α-ATP, α-ADP and NADH; and F, β-ATP [118]. Modified with permission from Elsevier.

**Figure 2:**
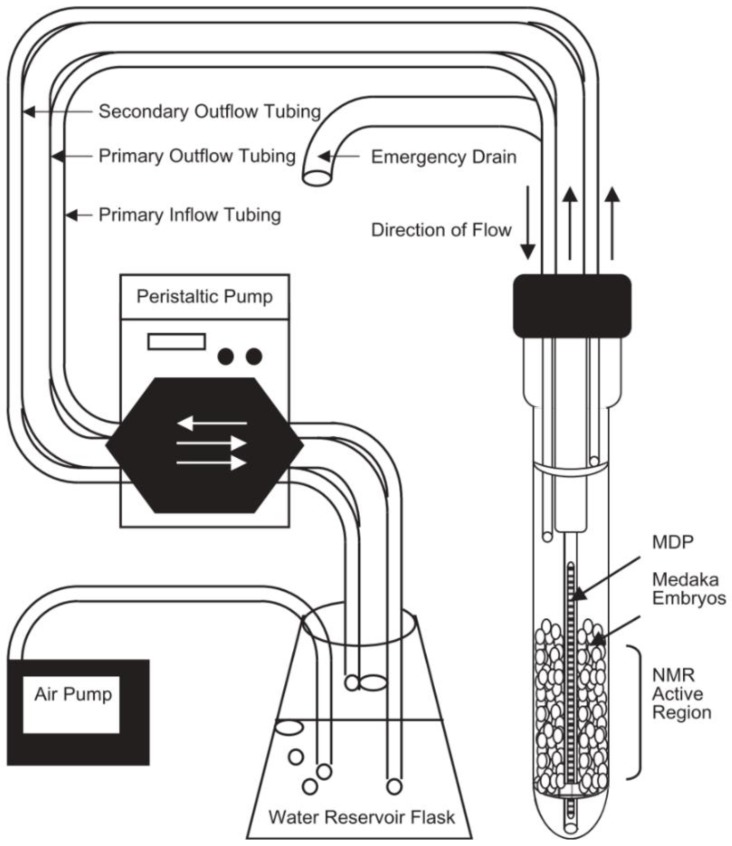
Example of one of the first flow systems for solution state NMR created by Pincetich et al. to keep Medaka embryos alive during the experiment in a 10 mm probe. Reproduced with permission from Elsevier [119].

Later an improved flow design was developed which enabled higher sensitivity and was used to deliver stressors to molluscs [117,118]. The results of the exposure can be seen in Figure 1. Further applications of NMR flow systems supplied organisms with oxygen and/or food, allowing them to be kept alive indefinitely [69,119,120]. This provides a low-stress environment thereby owing any metabolic changes detected as a direct response to the stressors, rather than a result of anoxic stress and/or starvation. An example of this flow system is shown in Figure 2. Japanese medaka embryos were studied using the flow system to deliver oxygenated water prior to exposing them to pesticide-treated water [119,120]. By monitoring their metabolic responses, a significant relationship between dose and response was observed [119,120]. The metabolic changes observed in these conditions correlated to traditional toxic endpoints, such as reduced growth and heart rates, abnormal development, and post-exposure mortality [120]. However, these studies were performed on a 10 mm probe which are not commonly available in NMR facilities [121].

More recently Soong et al. developed a 5 mm NMR flow system that allowed for metabolic profiling of isotopically ^13^C labelled freely swimming small organisms through high resolution 1D ^1^H and 2D ^1^H-^13^C NMR [69]. In this study, ^13^C labelled *D. magna* were placed in an NMR tube where oxygenated water was continuously circulated. The results of the flow, either on or off, were compared and anoxic stress was quickly observed in the absence of water circulation. The ability to obtain 2D ^1^H-^13^C NMR correlation experiments provides considerable spectral dispersion and permits a more comprehensive assignment of the metabolome in-vivo. Due to the key role *D. magna* play in environmental toxicity testing, Majumdar et al. chose them as the organism of interest in a recent publication of a standardized protocol for solution-state in-vivo NMR-based metabolomics studies [121]. In addition, flow systems can be applied to plant studies explored by Roscher et al. in a detailed guide on how to perform in-vivo analysis on plant materials, including considerations for appropriate plant material, culture medium, circulation systems, and data acquisition [122].

#### 2.1.2. Considerations

While solution-state NMR is a very useful technique and allows for NMR analysis in a low stress environment, there are a few challenges that need to be addressed. One is the low resolution obtained from 1D in-vivo NMR spectra, caused by magnetic susceptibility distortions (in simple terms the organisms body distorts the NMR magnetic field). This broadens the NMR signals causing signal overlap, and therefore, masking information from individual metabolites [94]. The simplest approach to overcome the spectral crowding is to disperse the NMR signal of interest into multiple dimensions. Novel approaches, and a more in-depth analysis will be discussed later.

Another challenge of ^1^H in-vivo NMR is the need to suppress the intense and broad water signal in aquatic organisms and their media. The ^1^H signal from water interfering with spectral resolution was once a major hurdle in solution-state in-vivo studies. Due to much of the sample being water, the signal is very broad and intense, masking important metabolite information and preventing full receiver optimization which in turn lowers sensitivity and reduces dynamics range. However, efficient water suppression methods (see later for further details) have allowed for more routine analysis using ^1^H in-vivo NMR.

### 2.2. High Resolution Magic Angle Spinning NMR

Although high resolution spectra can be acquired with solution-state NMR, information is only obtained on the truly dissolved metabolites. Species such as rigid gels and solids exhibit spectral broadening from dipolar interactions and anisotropy that make them challenging to detect without narrowing afforded by magic angle spinning. As such using solution-state NMR alone could lead to information from more rigid components such as membranes, cell walls, shells, and even bound stressors to be missed [123]. For this reason, it can be beneficial to compliment solution-state in-vivo NMR with gel-phase NMR spectroscopy, also known as high resolution-magic angle spinning (HR-MAS) NMR.

HR-MAS NMR probes allow the detection of solution and gel-like domains [124]. In HR-MAS NMR, the samples are spun at the magic angle of 54.74° to minimize the inhomogeneous broadening effects [125], while the presence of water in organisms help to reduce dipolar interactions that dominate in pure solids [126]. HR-MAS probes have a pulse field gradient, a lock channel and susceptibility matched stators. The result is that many modern solution-state NMR experiments can be implemented on HR-MAS probes and all components except true solids can be detected [127].

#### 2.2.1. In-Vivo Applications

**Figure 3:**
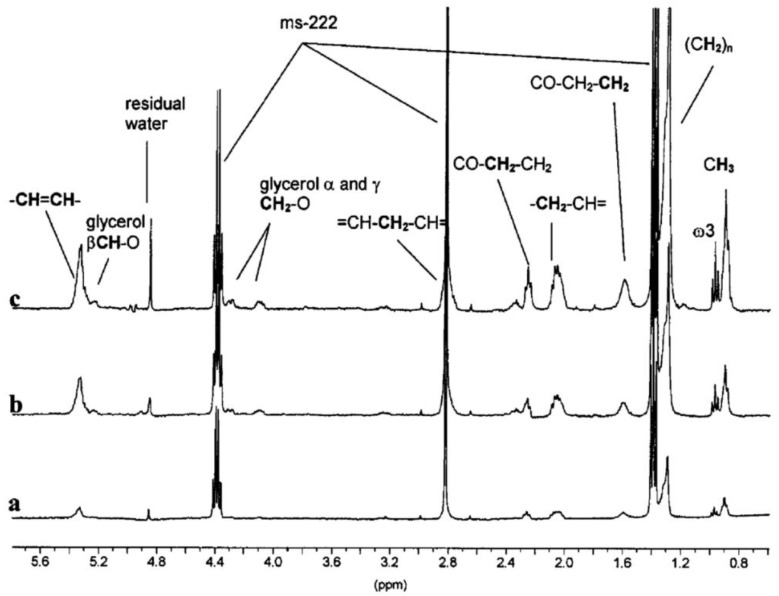
Example assigned spectra obtained from in-vivo proton high resolution-magic angle spinning (HR-MAS) NMR experiments examining *Daphnia magna* at: (**a**) 24 h old, (**b**) 7 days old with and (**c**) 7 days old without eggs in the brood pouch [129]. Reproduced with permission from Royal Society of Chemistry.

HR-MAS was first introduced in 1996, where researchers used a derivatized Wang resin (a linker for peptide synthesis) to demonstrate its application [124]. Since then, the approach has rapidly evolved to include in-vivo studies looking at a wide range of whole organisms, including *Drosophila melanogaster*, *Caenorhabditis elegans*, *Aporrectodea caliginosa*, *Calanus finmarchicus*, and *D. magna* where a range of well-resolved signals of metabolites were obtained (Figure 3) [50,128,129,130,131,132,133,134,135]. One study even applied a slow magic angle spinning approach to a whole live mouse [136]. Other studies have directed the technique towards plants leading to data on metabolic profiling during circadian cycles [137], carbon/nitrogen intracellular ratios [138], alkaloid metabolism [139], as well as assimilation of compounds and metabolite formation [140]. In-vivo samples, such as organisms and plants, can be studied with minimal to no sample preparation, since internal water acts as the solvent, allowing experiments to be done on samples in their natural state [141].

#### 2.2.2. Considerations

HR-MAS has demonstrated potential for in-vivo experiments, however, the major drawback to these studies is the unavoidable stress exerted from spinning. To determine the level of stress, Bunescu et al. tested the survival rates of *D. magna* following spinning under different speeds [129]. Their results showed that it was possible for the organisms to recover from certain experimental setups, especially when anesthetized. They then used these results to outline the optimal conditions for in-vivo HR-MAS NMR studies [129]. Additional improvements have been made by researchers that have focused on reducing the stress on organisms by slowing spinning through improvements of novel pulse sequences and suppression methods [94]. These results represent a promising future for in-vivo HR-MAS NMR experiments and permit studies without the use of anesthetic.

### 2.3. Comprehensive Multiphase NMR

**Figure 4:**
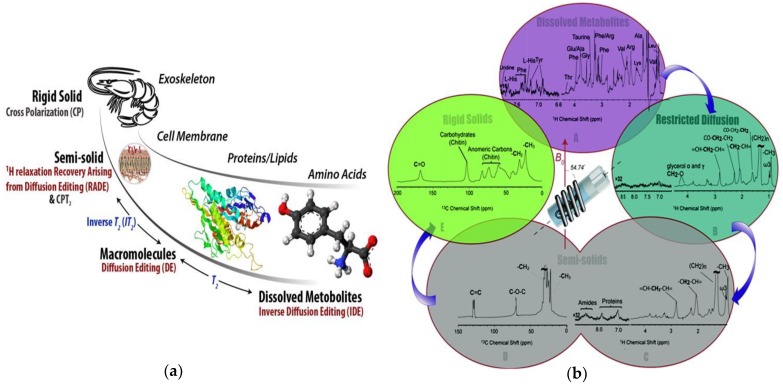
(**a**) An example of the types of information comprehensive multiphase (CMP)-NMR can provide when applied to a living freshwater shrimp; (**b**) Spectra from the metabolites (most mobile) through to the shell (most rigid) could be obtained in-vivo. Reproduced from Liaghati Mobarhan et al. under the Creative Commons Attribution 3.0 Unreported License [46].

In-vivo samples often encompass a range of phases, for example, in biological sections this may include liquid (blood), gel (tissue), and solid (shell/bone). It is the interactions between and the transport across these phases that ultimately give rise to the larger scale biological properties. As such it would be of great benefit to be able to study all components; liquid, gels and solids in-vivo. Although these phases can be individually studied using their respective probes (liquids, HR-MAS and solids NMR probes), very few groups have access to all three. In addition, using different probes provides data within individual phases, but lacks information on the interactions and kinetics occurring between the phases. As a solution, comprehensive multiphase (CMP) NMR spectroscopy was introduced in 2012 as a novel approach that combines a lock (for sharp lines), pulse field gradients (permits many 2D experiments and water suppression), magic angle spinning (narrows lines in gels/solids), and high power handling (required for pure solids) [127]. The technique allows for all bonds in all phases to be observed in natural, unaltered samples (see Figure 4), making it an ideal approach for materials with complex multiphase structures such as plants, air particles, sediments, and soils [56,66,97,98,142,143]. Using this approach, it has been possible to follow the penetration of contaminants into soil, as they move from solution into gel components, and finally become sequestered in the solid-phase [98]. The study was able to demonstrate the kinetic transfer between phases and identify the binding orientation and receptors in each phase, providing an in-depth insight into how and why the contaminant binds in soil. Further studies on soil have demonstrated that the approach can reveal how components organize and layer to form larger aggregate structures [97], critical information required for organisms in-vivo. Similarly, the technique applied in-vivo could be extremely important for understanding the binding and fate of contaminants and drugs [123]. In many ways it can be considered that metabolomics provides information on the rapid response of an organism to stress, whereas changes to the structural components may occur slower over time and reflect the longer-term impacts. An example, would be the altered composition in crustacean shells in polluted sites [144]. With the added ability to detect true solids, compared to HR-MAS probes, CMP-NMR probes can access all toxic impacts from the soluble metabolome through to the rigid exoskeleton, providing the potential for a complete insight into both long and short term toxic impacts.

#### 2.3.1. In-Vivo Applications

In some of its earliest applications, CMP-NMR technology has been applied to identify the complete metabolic and structural profile of intact ^13^C-labelled seeds [142]. This was followed by a second study which focused on the growth of the seeds and followed all the components during germination and early stages of development [145]. In 2016, CMP-NMR was applied to the living organisms *H. azteca* (freshwater shrimp) [46], an organism commonly used in aquatic toxicity testing, that swims and burrows into sediments, thus providing information on both water and sediment contamination [146]. CMP-NMR was able to fully differentiate the various phases, providing information on the shell and membranes, as-well as identifying a wide range of metabolites [46]. In many ways CMP-NMR can be thought of as “changing NMR technology to match the sample, rather than changing the sample to suit a specific type of NMR analysis” [66].

#### 2.3.2. Considerations

CMP-NMR is a very powerful approach and offers a range of novel information on intact samples. The major hurdle for in-vivo analysis is the rapid spinning of the sample. In a 2016 study spinning at 2.5 KHz on *H. azteca*, it was noted that spinning itself causes slight changes in the metabolome, especially for the amino acid alanine [46]. More recent work has slowed the spinning to 500 Hz, which greatly reduced stress and increased survival time [48]. However, at this speed sidebands (spectral artifacts) dominate. Novel pulse sequences were presented to overcome these artifacts in 1D ^1^H NMR [48], but no solution has been found yet for obtaining 2D ^1^H-^13^C at lower spinning speeds (500 Hz), spectra which greatly increase spectral dispersion and aids assignment. 

## 3. Challenges and Solutions

### 3.1. Water Suppression

In-vivo aquatic organisms are comprised mostly of water and are surrounded by water during experiments inside the NMR. The signal from this water needs to be suppressed for two main reasons. Firstly, the large peak can be extremely wide at the base, masking a wide range of metabolites signals [147], and secondly, and arguably more importantly, the water requires suppressing in order for the NMR receiver to be set to the maximum. The NMR receiver is somewhat analogues to a tape recorder in that if the recording sensitivity is set too high, “loud” signals, such as the intense signal from water, will lead to distortions and corrupt data. To avoid this, the receiver gain must be reduced. However, by reducing the receiver gain, the sensitivity is also reduced and low concentration signals close to the noise are lost due to the limited dynamic range of the NMR receiver. As such, in the case of in-vivo samples it is critical to reduce the water signal below that of the sample signals, permitting a maximum receiver gain and therefore allowing the in-vivo organisms to be studied without compromise. 

The simplest type of water suppression is presaturation, which is easy to implement and incorporate into a range of pulse sequences [148]. Unfortunately, presaturation is ineffective in dealing will extremely large (and often) broad signals as is in the case of environmental and in-vivo samples [149]. A detailed study compared a wide range of water suppression sequences for environmental samples, concluding that a combination of shaped presaturation and W5 WATERGATE was the most effective, and in fact the only sequence that could suppress especially broad water signals [149]. The presaturation block using shaped pulses helps narrow the water signal, which is followed by two W5 blocks surrounded by gradient pairs. The W5 blocks invert all signals except the water, which is de-phased twice by the gradients. The result is excellent water suppression often below the spectrometer noise, but at the cost of loss of signal in the central window (under and adjacent to the water). The approach has been successfully applied to study a wide range of matrices including, water samples [149], Antarctic ice [150,151], soils and plant materials [147] environmental photochemistry [152], and living organisms [46,69,121,153]. In the authors’ experience SPR-W5-WATERGATE is currently the only effective solution for suppressing the extremely wide water signal encountered in living organisms and would be the recommended starting point for any group attempting in-vivo NMR.

### 3.2. Spectral Overlap

One of the largest challenges with in-vivo NMR is spectral broadening caused by magnetic susceptibility distortions [154,155]. Distinct parts of the organism (shell, cell walls, cell contents, membranes, etc.) all have slightly different magnetic susceptibilities’ (i.e., they all interact with the external magnetic field differently). This causes slight distortions in the magnetic field, which in turn causes spins in various parts of the sample to experience different magnetic fields and thus resonate at slightly different frequencies. The result is that the net signal from the whole sample is broadened and key splitting information is lost under a broad spectral profile. Essentially two solutions are possible to overcome the broad chemicals shift (1) spread the signals out into more than one dimension to create the dispersion required for metabolite assignments in-vivo or (2) remove the magnetic susceptibility distortions.

#### 3.2.1. ^1^H-^13^C Multidimensional NMR

**Figure 5:**
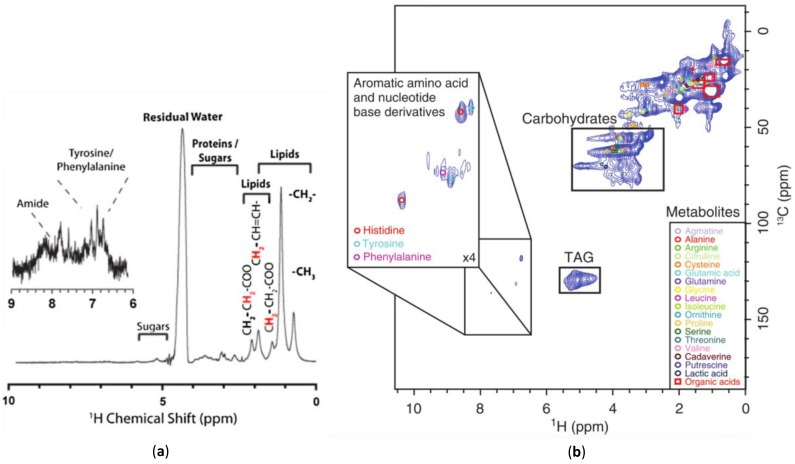
A comparison of: (**a**) a 1D ^1^H NMR spectrum [69]; (**b**) a 2D ^1^H-^13^C HMQC NMR spectrum [121] of ^13^C enriched *D. magna*. The additional spectral dispersion afforded by 2D permits metabolite assignments not possible from the 1D data. Modified with permission from John Wiley and Sons.

The simplest approach to increasing spectral dispersion and overcoming broad lines is to spread the signal into multiple dimensions. This can be achieved using ^1^H-^13^C HSQC NMR, which is relatively easy to obtain if the organisms are isotopically labelled with ^13^C to increase signal [21,156]. 1D ^1^H NMR has a peak capacity of 3000 peaks [157], whereas ^1^H-^13^C HSQC NMR approaches 2,000,000 providing the dispersion required for detailed metabolic assignments (see Figure 5) [80]. Modern HSQC (and HMQC) experiments use gradients for coherence selection, meaning a ^1^H that is not attached to a ^13^C is not selected. This results in the water signal being rejected by the gradient filter, allowing the experiments to have reasonable water suppression without modification. Furthermore, standard databases such as the Bruker Bioreference Databases [46,158,159] and the Human Metabolome Database [160,161,162,163] contain a wide array of assigned ^1^H-^13^C one bond correlations making assignments of HSQC relatively straight forward. Additional ^1^H-^1^H COSY data can be extremely useful to help confirm mixture assignments, which has been shown to be relatively easy to acquire under MAS conditions in-vivo. [46].

#### 3.2.2. Overcoming Magnetic Susceptibility Distortions

**Figure 6:**
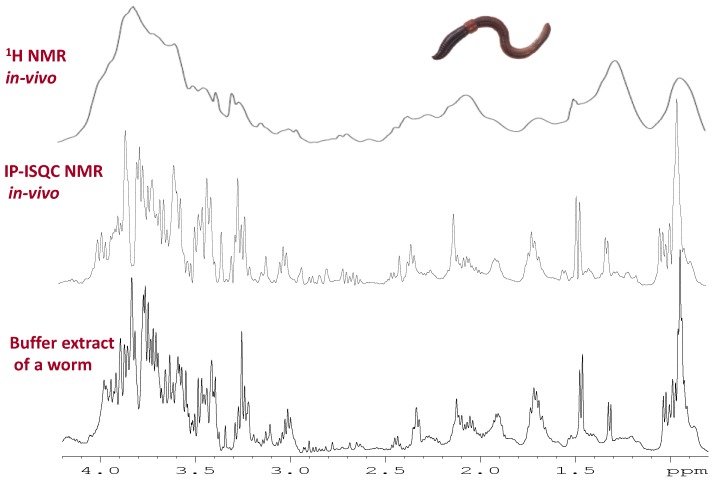
Example of an IP-iSQC NMR spectrum [153] which is used to remove magnetic susceptibility distortions from in-vivo NMR. The top spectrum shows a conventional ^1^H in-vivo NMR spectrum of a worm. ^1^H IP-iSQC approach which uses a 2D sequence to remove the distortions for the same worm (in-vivo). The bottom is a buffer extract from a similar worm (ex-vivo). The results demonstrate that the in-vivo IP-iSQC experiment produced near identical line shape, integral, and spectral profile as a buffer extract. Modified with permission from John Wiley and Sons.

The main approach to reduce or eliminate magnetic susceptibility distortions is to take advantage of intermolecular multiple quantum coherences between water and solutes. The approach was first discovered by Warren [164] and has been further developed by various other groups [165,166]. Long range interactions between water and solutes are reintroduced in the liquid state by utilizing a pulse field gradient. The interactions build up over distances longer than the more local susceptibility distortions and the chemical shift and magnetic susceptibility distortions can be separated by means of a 2D experiment. Recently, a phase sensitive version of the experiment was introduced and optimized for in-vivo samples with fast relaxation [153]. The resulting line shape from the In Phase—Intermolecular Single Quantum Coherence (IP-ISQC) experiment applied to living organisms was near identical to that obtained from buffer extracts (see Figure 6). The primary advantage of IP-ISQC is based on ^1^H detection, which does not require the use of ^13^C enriched organisms. As such the approach opens the possibility to study organisms directly from the environment rather than being reliant on lab raised ^13^C enriched organisms.

### 3.3. Sensitivity

NMR is a relatively insensitive technique. For larger organisms (>1 mm), it is not a major concern and signal is easy to detect, especially if numerous organisms are present in the sample. However, if small organisms such as eggs or individual organisms are to be studied then low amounts of biomass may make detection challenging or impossible using conventional 5 mm NMR probes. While cryoprobes can offer some improvement [167], microcoils hold greater potential for very small samples [168]. This was demonstrated in two recent studies by Fugariu et al. [81] and Grisi et al. [82]. Both studies demonstrate that small aquatic eggs could be studied. Grisi showed NMR on eggs as small as 100 pL, whereas Fugariu demonstrates analysis on the smallest coils (20 μm I.D.). These coils achieved a mass sensitivity improvement of >6000 times over a 5 mm room temperature probe, which translates into reduction in experimental time of ~36 million. These types of studies hold valuable potential for the analysis of resting eggs. Resting eggs are of great significance to environmental studies as they are produced by many planktonic organisms to ensure species survival during adverse times, and will exist in sediments up to decades until conditions improve [169]. Previous studies have indicated that eggs respond differently to toxins than young neonates [170]. Unfortunately, despite their critical role for the long-term survival of populations, very little is known about the impacts of toxic chemicals on resting eggs. Traditional toxicity tests are difficult to employ to study eggs, due to their lack of movement and reproduction, however, with the mass sensitivity improvement microcoil NMR has demonstrated, the technique is ideal for metabolic profiling in tiny samples. 

## 4. Why In-Vivo NMR?

### 4.1. Metabolic Pathways and Recovery

In-vivo NMR is highly complementary to conventional metabolic approaches. Traditional sampling approaches (homogenates of biofluids) provide a snapshot in time, whereas with in-vivo studies, the organisms are kept alive in the system, and data acquisition can occur continuously, and with high temporal resolution (sensitivity permitting). This provides potential to understand complex inter-connected response pathways to better understand how organisms react to, and deal with toxic chemicals. Furthermore, in-vivo NMR provides a convenient framework to study recovery from exposure [67]. If for example, an organism can return to homeostasis after exposure, then the contaminant likely only creates a temporary “flux response” from which the organism can fully recover. However, if the metabolome never returns to homeostasis post-exposure, this suggests permanent or long-term changes in the biochemistry which could be a precursor to disease or health deterioration [67]. A permanent change in the metabolome, even if it does not lead to rapid physical symptoms, is likely an important indicator for policy makers trying to estimate the safest, but also realistic targets for anthropogenic chemicals in the environment [67]. Studies with chemical mixtures could also provide information on synergistic effects. Some chemicals may not exhibit toxic properties alone, but when combined with others, can have deleterious effects. Continuous exposure to potentially hundreds of chemicals at trace concentrations is often the norm in the environment rather than the exception and the toxic synergism as well as impacts of long term exposure are currently not well understood [65,76]. 

### 4.2. Reducing the Impacts of Natural Variation

Traditional in-vitro metabolomics studies that use separate “control” and “exposed” populations have additional variation due the natural differences between individuals in the populations. This is unavoidable if the populations are sacrificed for analysis, as is the case for small organisms such as *C. elegans* and *D. magna* [102]. It has been shown this variance can be as much as 15% of the NMR signal [61]. This makes data analysis more challenging as statistical methods must detect tiny changes caused by the contaminant in the presence of often larger random variances. In-vivo NMR offers the potential to reduce this genetic variation by using the same organisms as the control and exposed populations [94]. For example, organisms could be placed inside the NMR and studied for six hours, after which they are then exposed. Data collected during the first six hours can be used as the control, and data after this point would contain additional signatures from the exposure. Care would have to be taken to ensure that the only changes are from the contaminant, thus the organisms must be supplied oxygenated water and food to avoid complications from starvation and anoxic stress. 

### 4.3. The Contaminant, Drug, or Nutrient

In-vivo NMR holds the potential to study not just the impacts of a contaminant, but if at high enough concentration, the chemical itself. Such studies should provide unique insights into binding mechanisms, biotransformation, bioaccumulation, and excretion. Experiments such as saturation transfer difference (STD) NMR can identify how molecules bind to receptors [171,172], whereas reverse-STD can identify which receptors do the binding [173,174]. If these approaches are combined with solid-state cross polarization then molecules that become sequestered in the most rigid components (shell, bone) can be selectively identified [98]. While studies have combined all these approaches to follow contaminant penetration into soil [98], they have yet to be applied in-vivo. The biggest hurdle at present is that many of the experiments are relatively insensitive, and contaminants are often present at low concentrations. However, the approach could have enormous impacts for understanding how nutrients (such as vitamins, phosphorous, etc.) are incorporated into living systems which are often present at much higher concentrations in-vivo.

### 4.4. Selective Isotopic Enrichment

Recent studies have used ^13^C enriched organisms to increase the signal in ^1^H-^13^C 2D NMR. This is an excellent approach as it makes metabolites in organisms easier to detect and ideal for non-targeted analysis. However, if the questions are more defined, for example “how does chemical X impact the Krebs cycle?”, it may be possible to selectively enrich precursor molecules to target pathways and extract higher resolution information on specific mechanisms [175].

In other cases, a heteronucleus specific to the contaminant, but not abundant in nature, could be highly beneficial. The most obvious nucleus being ^19^F as it is present in many environmental contaminants and drugs [40,176,177,178], and is a highly sensitive NMR nucleus. Perfluorinated compounds are stable in the environment and are absorbed into the body irreversibly. They have been found globally and are linked to reduced fertility, reduced birth weight, and changes in thyroid hormone levels along with many other negative consequences [67,179]. Other options for isotopic labelling may include the use of ^2^H in organic molecules or isotopic enrichment of heavy metals such as ^113^Cd, ^207^Pb, and ^199^Hg such that they are easier to detect and monitor in-vivo.

## 5. Conclusions

In-vivo NMR can provide a unique molecular-window into a living organism and its toxic response processes. The field of toxicity testing is changing rapidly, and with continuous chemicals entering the environment more reliable, and complementary techniques to study reaction mechanisms will be important to examine the impact on aquatic species, plants, and humans. While in-vivo NMR using high field instruments and flow systems were explored as early as the 80’s [110], it has not become routine due to complications from the intense water peak, broad signals, and low sensitivity. Using modern approaches including improved water suppression, isotopic enrichments, cryoprobes, micro-coils, and multidimensional NMR, many of these hurdles are being overcome and in the last few years in-vivo NMR is making a resurgence as a very powerful technique with immense potential. A considerable amount of work is still required, including hardware (improved coils and flow cells), experiments (targeted and higher order multidimensional experiments to extract more information), and data processing (extracting key information quickly from massive amounts of data). Both smaller detection technologies (microcoils for single cell and egg analysis) and larger bore magnets for larger organisms need to be explored. Furthermore, the potential of in-vivo low field NMR is an exciting future prospect. While low field NMRs suffer from reduced resolution and sensitivity, their low cost and portability make them ideal for field deployment with often nothing more than a power supply required for monitoring. Some examples, include a mobile NMR lab for leaf phenotyping in the field [180], a portable sensor for monitoring water and sap flow [181], a device to detect water content in trees [182,183], fast field cycling NMR in plant leaves [184], as-well as lipid and metabolites profiling in seeds [185]. Advancement in technologies such as dynamic nuclear polarization in combination with low field NMR [186], and zero/ultra-low field NMR with optical detection [187] offer potential for huge increases in sensitivity that would advance low field NMR into a powerful tool for in-vivo analysis and monitoring. 

In summary, given NMR’s ability to examine inside a living organism and provide unprecedented information on the living metabolome, in-vivo NMR will undoubtedly evolve to become a central and key tool for understanding living processes and how they are impacted by toxic chemicals.
